# High resolution melting analysis of *KRAS*, *BRAF* and *PIK3CA* in *KRAS* exon 2 wild-type metastatic colorectal cancer

**DOI:** 10.1186/1471-2407-13-169

**Published:** 2013-04-01

**Authors:** Joana G Guedes, Isabel Veiga, Patrícia Rocha, Pedro Pinto, Carla Pinto, Manuela Pinheiro, Ana Peixoto, Maria Fragoso, Ana Raimundo, Paula Ferreira, Manuela Machado, Nuno Sousa, Paula Lopes, António Araújo, Joana Macedo, Fernando Alves, Camila Coutinho, Rui Henrique, Lúcio L Santos, Manuel R Teixeira

**Affiliations:** 1 Departments of Genetics, Portuguese Oncology Institute, Porto, Portugal; 2 Departments of Oncology, Portuguese Oncology Institute, Porto, Portugal; 3 Departments of Pathology, Portuguese Oncology Institute, Porto, Portugal; 4 S. Sebastião Hospital, Santa Maria da Feira, Portugal; 5 Trás-os-Montes e Alto Douro Hospital Center, Vila Real, Portugal; 6 Alto Ave Hospital Center, Guimarães, Portugal; 7 Departments of Surgery, Portuguese Oncology Institute, Porto, Portugal; 8 Abel Salazar Biomedical Sciences Institute (ICBAS), University of Porto, Porto, Portugal

## Abstract

**Background:**

KRAS is an EGFR effector in the RAS/RAF/ERK cascade that is mutated in about 40% of metastatic colorectal cancer (mCRC). Activating mutations in codons 12 and 13 of the *KRAS* gene are the only established negative predictors of response to anti-EGFR therapy and patients whose tumors harbor such mutations are not candidates for therapy. However, 40 to 60% of wild-type cases do not respond to anti-EGFR therapy, suggesting the involvement of other genes that act downstream of EGFR in the RAS-RAF-MAPK and PI3K-AKT pathways or activating *KRAS* mutations at other locations of the gene.

**Methods:**

DNA was obtained from a consecutive series of 201 mCRC cases (FFPE tissue), wild-type for *KRAS* exon 2 (codons 12 and 13). Mutational analysis of *KRAS* (exons 3 and 4), *BRAF* (exons 11 and 15), and *PIK3CA* (exons 9 and 20) was performed by high resolution melting (HRM) and positive cases were then sequenced.

**Results:**

One mutation was present in 23.4% (47/201) of the cases and 3.0% additional cases (6/201) had two concomitant mutations. A total of 53 cases showed 59 mutations, with the following distribution: 44.1% (26/59) in *KRAS* (13 in exon 3 and 13 in exon 4), 18.6% (11/59) in *BRAF* (two in exon 11 and nine in exon 15) and 37.3% (22/59) in *PIK3CA* (16 in exon 9 and six in exon 20). In total, 26.4% (53/201) of the cases had at least one mutation and the remaining 73.6% (148/201) were wild-type for all regions studied. Five of the mutations we report, four in *KRAS* and one in *BRAF*, have not previously been described in CRC. *BRAF* and *PIK3CA* mutations were more frequent in the colon than in the sigmoid or rectum: 20.8% *vs.* 1.6% *vs.* 0.0% (*P=*0.000) for *BRAF* and 23.4% *vs.* 12.1% *vs.* 5.4% (*P*=0.011) for *PIK3CA* mutations.

**Conclusions:**

About one fourth of mCRC cases wild-type for *KRAS* codons 12 and 13 present other mutations either in *KRAS*, *BRAF*, or *PIK3CA*, many of which may explain the lack of response to anti-EGFR therapy observed in a significant proportion of these patients.

## Background

The increasing knowledge of cancer biology has led to the development of targeted therapies, designed to interfere with specific molecules involved in tumor growth and progression [1,2]. EGFR is a transmembrane receptor tyrosine kinase (TK) implicated in several cellular responses, like apoptosis, differentiation, cellular migration, and adhesion. This TK and the pathways it controls play an important role in colorectal carcinogenesis [3-5], making it a good target for biological therapy of this disease [2]. A network of various signal transduction cascades is stimulated by EGFR signaling, namely the RAS/RAF/MEK/ERK, PI3K/AKT, JAK/STAT and PLCγ pathways. Cetuximab, a human-mouse chimeric IgG1, and panitumumab, a fully human IgG2, are monoclonal antibodies (moABs) that compete with EGFR’s ligands and specifically bind to the receptor, blocking ligand-induced downstream signaling [2]. These targeted agents have been evaluated in several clinical trials for the treatment of metastatic colorectal cancer (mCRC), either alone, in combination with fluoropyrimidine-based chemotherapy regimens, or with bevacizumab [6-11], and have subsequently been approved by the European Medicines Agency (EMEA) and the U.S. Food and Drug Administration (FDA).

Several retrospective analyses of *KRAS* mutational status in tumors from patients treated with cetuximab and panitumumab found an association between *KRAS* codons 12 or 13 activating mutations and lack of treatment efficacy [6-11]. In normal cells, the KRAS protein alternates between an inactive GDP-bound form and an active GTP-bound form. Mutations in *KRAS* codons 12 and 13 originate a constitutively active protein, resulting in a continuous and self-sufficient (independent of ligand binding) KRAS signaling. These *KRAS* mutations, present in about 40% of mCRC, are the only available (negative) predictors of response to anti-EGFR moABs, and this therapy is strictly indicated for patients with *KRAS* wild-type mCRC [6,9,12,13]. However, absence of *KRAS* exon 2 mutations does not guarantee treatment response, as only 40 to 60% of these cases respond to anti-EGFR therapy [7,13,14]. Other mutations in genes encoding proteins that act downstream of EGFR, such as *KRAS*, *BRAF*, and *PIK3CA*, may be responsible for the absence of treatment response in such cases.

In this study, 201 cases of mCRC wild-type for *KRAS* codons 12 and 13 were screened for mutations in other potential biomarkers of response to anti-EGFR treatment, namely in the coding regions of KRAS switch II and G5 regions (exons 3 and 4), the P-loop and activation segment of BRAF (exons 11 and 15), and in PIK3CA’s helical and kinase domains (exons 9 and 20) [15,16].

## Methods

### Samples

A consecutive series of tumor samples (formalin-fixed and paraffin-embedded, wild-type for *KRAS* codons 12 and 13) from 212 patients with stage IV colorectal adenocarcinoma were retrospectively analyzed. These patients were referred to the Genetics Department of IPO-Porto, between August 2008 and January 2010, for routine *KRAS* codons 12 and 13 mutation analysis and were considered wild-type for both codons by at least two of four independent methods in a previous work by our group, representing 56.5% of the cases [17]. When patients received neoadjuvant radiotherapy, diagnostic tumor biopsies were used for mutation analyses instead of primary tumors. Of these 212 cases, eight were excluded due to lack/poor quality DNA and another three because of missing clinical data. A total of 201 cases were analyzed and their clinical characteristics are listed in Additional File 1: Table S1. This study was approved by the Institutional Review Board of the Portuguese Oncology Institute-Porto and written informed consent was obtained from all patients before testing.

### DNA extraction

Hematoxylin and eosin (H&E) stained slides from tumors of each case were reviewed by a pathologist, who delimited areas containing at least 70% tumor cells. Unstained slides were immersed in xylene for 5 minutes and twice in ethanol 100% for 5 minutes. Tumor areas were then delimited, by comparison with correspondent H&E stained slides, and macrodissected. DNA was isolated from scrapped material using the methods described by Lungu *et al.*[18], phenol-chlorophorm [19], or by the QIAamp® DNA FFPE TissueKit (QIAGEN, Hilden, Germany). DNA was quantified by spectrophotometry with NanoDrop ND-1000® (Thermo Fisher Scientific Inc., Waltham, MA, USA).

### Mutational analysis

We searched for mutations in *KRAS* mutational hotspots other than exon 2 (NM_004985.3; exons 3 and 4), as well as in *BRAF* (NM_004333.3; exons 11 and 15), and in *PIK3CA* (NM_006218.2; exons 9 and 20). High resolution melting (HRM) was used as a screening method to distinguish mutated from wild-type samples. DNA sequencing of one strand was performed in those samples considered positive by HRM. All mutated samples were subject to a second HRM and DNA sequencing analyses in order to validate the results.

PCR amplification and HRM were performed on a LightCycler® 480 II Real-Time System (Roche Diagnostics, Basel, Switzerland). PCR mastermix containing one primer pair, all PCR reagents, and DNA (Additional File 2: Table S2) was added to each well of a 96 well plate. Fifteen microliters of mineral oil were added to all wells in order to prevent evaporation and cross-contamination. Plates were sealed with sealing film and centrifuged at 2000 rpm for 2 minutes. All samples were run in duplicate.

Primer pairs for *KRAS* exons 3 and 4 were designed with primer-BLAST software (http://www.ncbi.nlm.nih.gov/tools/primer-blast/; Additional File 3: Table S3). Primer pairs for *PIK3CA* exons 9 and 20 and *BRAF* exons 11 and 15 were previously described [20-22]. Cycling and melting conditions were as follows: an initial denaturation at 95°C for 10 minutes followed by 40 cycles of 20 seconds at 90°C, 20 seconds at 67°C, and 20 seconds at 72°C (for *PIK3CA* exons 9 and 20, *BRAF* exon 11, and *KRAS* exon 3) or 40 cycles of 20 seconds at 95°C, 20 seconds at 65°C, and 20 seconds at 72°C (for *BRAF* exon 15 and *KRAS* exon 4) and a final extension at 72°C for 10 minutes. One heteroduplex cycle was done at 95°C for 5 minutes and 40°C for 1 minute, followed by melting from 70°C to 90°C with 25 acquisitions/°C and a 1 minute cooling to 40°C with a ramp rate of 2.2°C/second.

Amplification and melting curves were generated and analyzed using the LightCycler® 480 Gene Scanning software version 1.5 (Roche diagnostics). Samples with late amplification were excluded from the analysis. PCR amplification products generated by the LightCycler PCR were purified using illustra GFX PCR DNA and Gel Band Purification Kit (GE Healthcare, Little Chalfont, UK) according to the manufacturer’s protocol. For the sequencing reaction, 1 μL of purified PCR amplification products were used with 1 μL of Big Dye® Terminator V1.1 cycle sequencing Ready Reaction Mix (dNTPs. ddNTPs-fluorocromes, MgCl_2_, Tris–HCl buffer), 1.9 μL of Big Dye® Terminator V1.1, V1.3 5x sequencing buffer (Applied Biosystems Inc., Fostercity, CA, USA), 350 nM of primers described above and bidestilled sterile water to a total volume of 10 μL. The sequencing reaction consisted of an initial denaturation step at 96°C for 5 minutes, followed by 35 cycles of 96°C for 10 seconds, 52°C for 5 seconds and 60°C for 4 minutes. Sequencing reaction products were purified prior to sequencing in order to remove contaminants, using illustra Sephadex® G-50 fine (GE Healthcare Life Sciences). After purification, 12 μL of Hi-Di™ Formamide (Applied Biosystems) were added to the sequencing product. Sequencing PCR products were run on an ABI PRISM™ 310 Genetic Analyzer and the respective electropherograms were analyzed with Sequencing Analysis Software v5.2 (Applied Biosystems). All electropherograms were read manually.

### Statistical analysis

Chi-square or Fisher’s exact tests were performed as appropriate to assess statistical differences between two groups of patients, and linear-by-linear association was used when comparing more than two sequential groups. Associations were considered statistically significant when P≤0.05. All statistical analyses were performed with SPSS Statistics v.19 (SPSS Inc., IL, USA).

## Results

### Mutation frequencies

**Figure 1:**
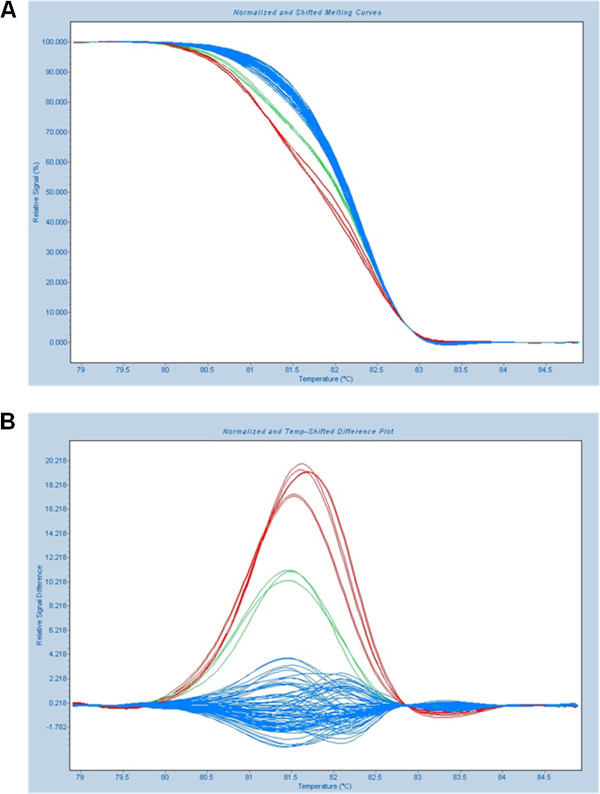
**High resolution melting analysis of *PIK3CA *exon 9. A**) Normalized and **B**) difference graph, containing wild-type samples (blue) and mutated samples (green and red).

A total of 201 *KRAS* exon 2 wild-type mCRC samples were screened by HRM for mutations in exons 3 and 4 of *KRAS*, exons 11 and 15 of *BRAF,* and exons 9 and 20 of *PIK3CA* (Figure 1). Subsequent automated sequencing of HRM positive cases confirmed the presence of 59 mutations in 53 cases, with the following distribution: 44.1% (26/59) in *KRAS* (13 in exon 3 and 13 in exon 4), 18.6% (11/59) in *BRAF* (two in exon 11 and nine in exon 15) and 37.3% (22/59) in *PIK3CA* (16 in exon 9 and six in exon 20). One mutation was present in 23.4% (47/201) of the cases and 3.0% additional cases (6/201) had two concomitant mutations. In total, 26.4% (53/201) of the cases had at least one mutation and the remaining 73.6% (148/201) were wild-type for all regions studied. All mutations were found in heterozygosity and were confirmed in a second HRM and DNA sequence analysis.

Samples with single mutations were distributed as follows: 10.0% (20/201) were *KRAS* mutated, 5.0% (10/201) in exon 3 and 5.0% (10/201) in exon 4; 5.0% (10/201) had one *BRAF* mutation, 0.5% (1/201) in exon 11 and 4.5% (9/201) in exon 15; 8.5% (17/201) were *PIK3CA* mutated, 7.0% (14/201) in exon 9 and 1.5% (3/201) in exon 20 (Table 1).

**Table 1:** **Frequency of *KRAS*, *BRAF *and *PIK3CA *single mutants (N=47/201)**

	**Mutation**		**No. of cases**	**Total no. of cases (%)**
**Gene and exon**	**cDNA sequence**	**Protein sequence**		
*KRAS* exon 3	c.151_195dup	p.Cys51_Ser65dup	1	10 (5.0%)
	c.176_178del	p.Ala59del	1	
	c.179G>T	p.Gly60Val	1	
	c.181C>A	p.Gln61Lys	1	
	c.182A>T	p.Gln61Leu	3	
	c.182A>G	p.Gln61Arg	2	
	c.183A>C	p.Gln61His	1	
*KRAS* exon 4	c.436G>A	p.Ala146Thr	10	10 (5.0%)
*BRAF* exon 11	c.1397G>A	p.Gly466Glu	1	1 (0.5%)
*BRAF* exon 15	c.1799 T>A	pVal600Glu	8	9 (4.5%)
	c.1808A>G	p.Lys601Glu	1	
*PIK3CA* exon 9	c.1624G>A	p.Glu542Lys	5	14 (7.0%)
	c.1633G>A	p.Glu545Lys	5	
	c.1635G>T	p.Glu545Asp	1	
	c.1636C>A	p.Gln546Lys	3	
*PIK3CA* exon 20	c.3129G>A	p.Met1043Ile	1	3 (1.5%)
	c.3140A>G	p.His1047Arg	1	
	c.3140A>T	p.His1047Leu	1	

**Figure 2:**
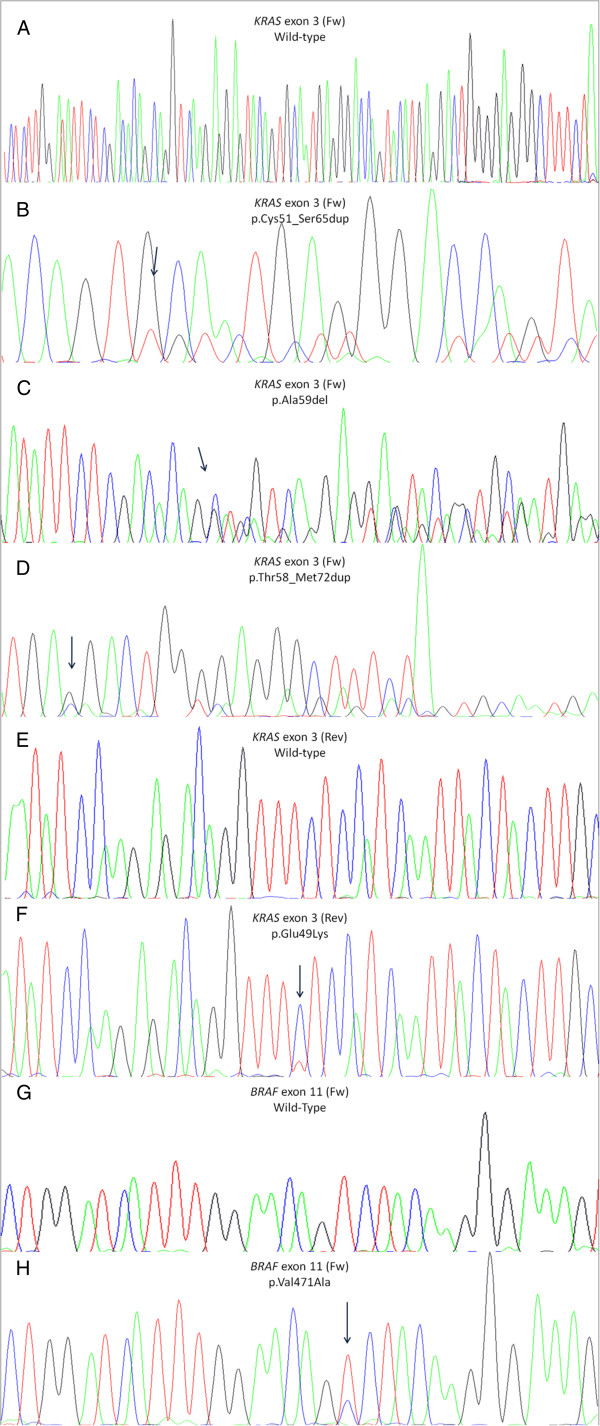
**Electropherograms of the novel mutations found in this series and of wild-type samples. A**) and **E**) *KRAS* exon 3 wild-type samples; **B**), **C**), **D**) and **F**) *KRAS* exon 3 mutations; **G**) *BRAF* exon 11 wild-type; **H**) *BRAF* exon 11 mutant sample. Fw: forward strand. Rev: reverse strand. Arrow indicates the mutational spot.

Concomitant mutations were found with the following distribution: 2.0% (4/201) of cases had simultaneous mutations in *PIK3CA* and *KRAS*, 0.5% (1/201) in *PIK3CA* and *BRAF*, and 0.5% (1/201) two mutations in *KRAS* (Table 2). Of all mutations here reported, five have not been previously described in colorectal cancer [23-25]: two duplications, one deletion, and one point mutation in *KRAS* and one point mutation in *BRAF* (Figure 2).

**Table 2:** Frequency of double mutants (N=6/201)

	**Coexisting mutations**		**No. of cases**	**Total no. of cases (%)**
**Gene and exon**	**cDNA sequence**	**Protein sequence**		
*PIK3CA* exon 9	c.1633G>A	p.Glu545Lys	1	6 (3.0%)
*KRAS* exon 4	c.436G>A	p.Ala146Thr		
*PIK3CA* exon 9	c.1624G>A	p.Glu542Lys	1	
*KRAS* exon 3	c.183A>C	p.Gln61His		
*PIK3CA* exon 20	c.3139C>T	p.His1047Tyr	1	
*KRAS* exon 3	c.173_217dup	p.Thr58_Met72dup		
*PIK3CA* exon 20	c.3140A>G	p. His1047Arg	1	
*KRAS* exon 4	c.436G>A	p.Ala146Thr		
*PIK3CA* exon 20	c.3140A>G	p.His1047Arg	1	
*BRAF* exon 11	c.1412 T>C	p.Val471Ala		
*KRAS* exon 3	c.145G>A	p.Glu49Lys	1	
*KRAS* exon 4	c.436G>A	p.Ala146Thr		

### KRAS mutations

The eight *KRAS* codon 61 mutations present in our series result in four different amino acid substitutions (p.Gln61His, p.Gln61Lys, p.Gln61Leu, and p.Gln61Arg), with the p.Gln61Leu being the most frequent codon 61 mutation in this series (37.5%; 3/8). Mutations found in codon 146 were restricted to the p.Ala146Thr substitution.

Besides those in codons 61 and 146, other *KRAS* exon 3 mutations represented 19% (5/26) of all *KRAS* changes, including one deletion (p.Ala59del), two point mutations (p.Glu49Lys and p.Gly60Val) and two large in frame duplications (p.Cys51_Ser65dup and p.Thr58_Met72dup). Of these five mutations, only p.Gly60Val has been recently reported in CRC [26], whereas the other four are novel mutations in CRC [23].

One of the cases carrying a *KRAS* p.Gln61His mutation had a concomitant *PIK3CA* p.Glu542Lys substitution. The p.Thr58_Met72dup duplication occurred in one tumor carrying a *PIK3CA* p.His1047Tyr substitution. Two tumors with *KRAS* Ala146Thr mutations also had a *PIK3CA* mutation, either p.Glu545Lys or p.His1046Arg. In addition, one case harbored two *KRAS* mutations, namely p.Glu49Lys and p.Ala146Thr.

### BRAF mutations

The frequency of *BRAF* p.Val600Glu mutations found in this series was 4.0% (8/201). This mutation represented 89% (8/9) of exon 15 mutations and 73% (8/11) of all *BRAF* mutations. We also found mutations in codons 601 (p.Lys601Glu), 466 (p.Gly466Glu), and 471 (p.Val471Ala) of *BRAF*, the latter not previously described in mCRC [23]. One *BRAF* mutation, p.Val471Ala, occurred in a tumor also carrying a *PIK3CA* p.His1047Arg mutation.

### PIK3CA

*PIK3CA* mutations were present in 10.9% (22/201) of the tumors, unequally distributed between exons 9 and 20: 73% (16/22) were helical domain mutants (p.Glu542Lys, p.Glu545Lys, p.Glu545Asp, and p.Gln546Lys) and 27% (6/22) kinase domain mutants (p.Met1043Ile, p.His1047Arg, p.His1047Leu, and p.His1047Tyr). Five of the *PIK3CA* mutants also contained another mutation in either *KRAS* or *BRAF*.

### Clinicopathological associations

*KRAS* mutations were more frequent in patients older than the median age of diagnosis (21.5% *vs.* 8.2%; *P*=0.034), whereas no statistically significant differences were found for *BRAF* or *PIK3CA* mutations regarding this parameter.

**Figure 3:**
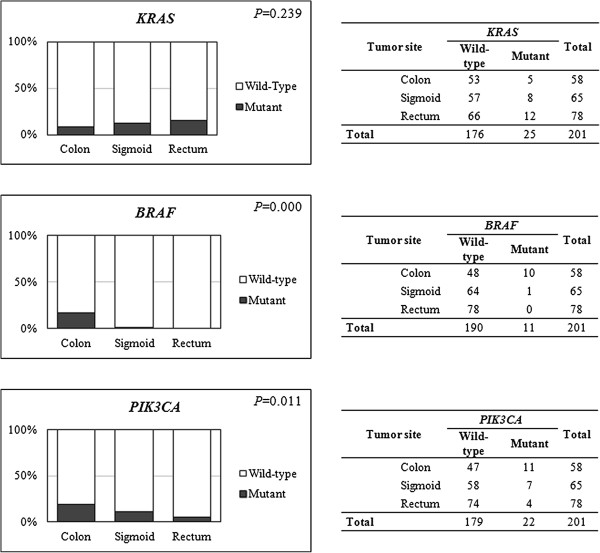
**Distribution of *KRAS*, *BRAF *and *PIK3CA *mutations according to primary tumor site.**

*BRAF* and *PIK3CA* mutations were more frequent in the colon than in the sigmoid or rectum: 20.8% *vs.* 1.6% *vs.* 0.0% (*P=*0.000) for *BRAF* and 23.4% *vs.* 12.1% *vs.* 5.4% (*P*=0.011) for *PIK3CA* mutations (Figure 3). Although the frequency of *KRAS* mutations is higher in sigmoid and rectum, the difference is not statistically significant. No significant differences were found between genders regarding *KRAS*, *BRAF*, or *PIK3CA* mutation frequencies.

## Discussion

We here show that more than one-fourth of *KRAS* exon 2 wild-type mCRC patients present other mutations in *KRAS*, *BRAF,* and/or *PIK3CA* and report five novel mutations, namely four in *KRAS* exon 3 and one in *BRAF* exon 11. The mutational frequencies in the most commonly altered codons of *KRAS*, *BRAF,* and *PIK3CA* genes were in accordance to those previously described in the literature. We have previously shown that HRM is a highly sensitive technique to detect mutations in mCRC, being significantly more sensitive and cheaper than standard Sanger sequencing [17], combining high sensitivity with the ability to detect novel mutations. A *KRAS* codon 61 and 146 mutation frequency of 10.4% is similar to what has been reported for *KRAS* codons 12 and 13 wild-type patients (6.5% - 10.5%) [24,25]. The frequency of the *BRAF* p.Val600Glu mutation was lower (4.0%) but comparable to the reported frequency range of 4-18% in mCRC without *KRAS* codon 2 mutations [25,27-30]. Since the *BRAF* p.Val600Glu mutation is associated with microsatellite instability (MSI) status and right colon tumors [31-33], variations in sample characteristics between studies can account for the wide frequency range, but this is often hard to verify as many studies in mCRC do not describe the primary tumor localization nor MSI status. *PIK3CA* mutations were present in 10.9% of the tumors, which is similar to previous reports [24,34-36]. Interestingly, both *BRAF* (*P*=0.000) and *PIK3CA* (*P*=0.011) mutations were significantly more frequent in colon than in sigmoid or rectal carcinomas. On the other hand, an association was found between *KRAS* mutations and older age at diagnosis (*P*=0.034), which was not observed for *BRAF* or *PIK3CA*. These findings should be confirmed in larger series in order to evaluate its significance.

*KRAS* codon 61 oncogenic mutations occur at an essential position for GTP hydrolysis and decrease RAS-mediated GTP hydrolysis [37], resulting in transformation efficiencies that vary up to 1000-fold [38]. It has been demonstrated *in vivo* that codon 61Leu, Lys, and Arg induce a strong oncogenic phenotype, whereas 61 His is a moderately transforming mutant [38]. Aminoacid Ala146 is involved with guanine base interaction and mutations in this codon do not affect GTPase activity, but are associated with an increased GDP to GTP exchange. Expression of p.Ala146Thr mutations *in vivo* results in elevated RAS-GTP and phosphorylated ERK compared to wild-type *KRAS*, albeit lower than that caused by codon 12 mutations [39]. However, there is no data available to determine the influence in RAS protein structure of the novel deletion (p.Ala59del) and the two novel large in frame duplications (p.Cys51_Ser65dup and p.Thr58_Met72dup) we found in exon 3, but the fact that they are located in the switch II region is an indicator that they may activate RAS by impairing GTP hydrolysis. Of notice, few *KRAS* duplications and deletions have been reported: only three in exon 2 and two in exon 3. No functional studies exist regarding the role of *KRAS* p.Gly60Val or p.Glu49Lys mutations, but it is known that the Gly60 residue interacts with γ-phosphate of GTP and is a conserved amino acid in the superfamily of GTPases [40], facts that argue in favor of Gly60Val pathogenicity.

Both *BRAF* p.Val600Glu and p.Lys601Glu mutations occur in the activation site and originate proteins with high kinase activity. *In vitro*, *BRAF* p.Val600Glu and p.Lys601Glu proteins have higher TK activity than the wild-type protein (500-fold and 120-fold higher, respectively) [41]. Mutants p.Val600Glu also show a six-fold higher ERK signaling *in vivo*, when compared to the wild-type protein [41]. These high TK activity mutants are thought to simulate the conformational changes caused by activation segment phosphorylation, resulting in protein ligand-independent constitutive activation. On the other hand, the Gly466 is the second glycine of the P-loop GXGXXG motif (G=glycine; X=variable), conserved in more than 99% of all kinases [15]. It is an important catalytic residue and its substitution to glutamic acid (p.Gly466Glu) originates a protein with higher ERK signaling than wild-type BRAF but a diminished, although constitutively active, TK activity [41]. It has been proposed that increased ERK signaling occurs via an association between BRAF and CRAF and their ability to stimulate ERK is dependent on CRAF activation [41]. It has been demonstrated that p.Gly466Glu cells depend on CRAF for ERK signaling: they induce strong CRAF activation and CRAF depletion significantly suppresses ERK signaling [41].

*PIK3CA* helical domain mutants p.Glu542Lys, p.Glu545Lys, and p.Gln546Lys and kinase domain mutants p.Met1043Ile, p.His1047Arg, p.His1047Leu, and p.His1047Tyr all display enhanced lipid kinase activity compared to the wild-type p110α, and p.Glu542Lys, p.Glu545Lys, and p.His1047Arg induce AKT phosphorylation at higher levels than the normal protein [42-48]. Furthermore, p.Glu545Lys and p.His1047Arg promote cell growth and invasion in CRC cell lines, and mutations p.His1047Leu and p.His1047Tyr induce oncogenic transformation in primary cell cultures of chicken embryo fibroblasts [44,46]. In CRC, Met1043 is less frequently altered than His1047 (0.8% *vs* 7.1%) [49]. Amino acids 1043 and 1047 are located on the same protein helix and probably affect protein function by altering activation loop conformation, leading to elevated kinase activity [42,50]. The above referred helical domain mutations occur at residues involved in the interaction with the adaptor protein and are thought to abrogate its inhibitory effect by increasing the positive charge on the surface of the helical domain. It has also been demonstrated that p85 does not inhibit these mutants *in vitro*[45,50]. Finally, the Glu to Asp substitution in codon 545 has not been functionally studied, but both amino acids involved are polar and negatively charged, thus making it unlikely that this substitution will produce the same effect on p110α as those described above. In this study we also observed that *PIK3CA* codon 545 substitutions account for 9.8% of *PIK3CA* mutations in CRC [49] and, since the carcinoma carrying the *PIK3CA* p.Glu545Asp mutation did not present mutations in either *KRAS* or *BRAF*, it is conceivable that this mutation confers some selective advantage.

In six cases, we found two different mutations in the various exons studied, most commonly coexistence of a *PIK3CA* mutation with either a *KRAS* or a *BRAF* mutation. Coexisting mutations of *KRAS*/*BRAF* and *PIK3CA* have been reported in several studies [24,30,31,36], with *PIK3CA* exon 20 mutations more frequently co-occurring with mutations of unknown significance or with *KRAS* codon 146 mutations [24]. Additionally, we found one instance of coexistence of the *PIK3CA* p.His1047Arg mutation with the novel mutation *BRAF* p.Val471Ala, a conserved residue of RAF proteins, having no functional studies available to allow inferences on its oncogenic potential. Finally, one case harbored two *KRAS* mutations, namely the novel p.Glu49Lys and the p.Ala146Thr mutations. The coexistence of two mutations in the same gene or in different genes may be explained by a synergistic contribution of both mutations to pathway activation or the occurrence of each mutation in different subclones as a result of tumor clonal divergence.

According to a recently published large multicentric study involving retrospective mutation analysis on *KRAS*, *BRAF*, *NRAS*, and *PIK3CA* in mCRC and the impact of mutations in these genes on cetuximab treatment efficacy [24], tumors with *KRAS* codon 61 mutations have lower response rates and *PIK3CA* exon 20 mutations are associated with a worse outcome after cetuximab treatment, with *NRAS* mutations (codons 12, 13 and 61) being predictive of nonresponse to this targeted therapy. On the other hand, this retrospective study indicates that *KRAS* codon 146 and *PIK3CA* codon 9 mutations do not affect cetuximab efficacy. This study also confirmed the inefficacy of cetuximab in patients with *BRAF* p.Val600Glu mutations, with response rates of 8% *vs* 38% for *BRAF* mutated and wild-type, respectively [24,25,27,28,51]. No associations with treatment response have been published for *BRAF* exon 11 mutations or any other *KRAS* exon 3 mutations besides those in codon 61, essentially because they are rare. We could not make an evaluation of the predictive value of these mutations in our patients at the time of writing due to the low number of mutated cases that have been treated with cetuximab, but in face of the findings of De Roock *et al.*[25] our mutation data indicates that at least 10.9% of our mCRC patients wild-type for *KRAS* codon 12 and 13 would not benefit from anti-EGFR targeted therapy. Further prospective or functional studies will be necessary to evaluate the predictive value of the remaining mutations, including the novel *KRAS* and *BRAF* mutations we here report.

## Conclusions

About one fourth of mCRC cases wild-type for *KRAS* codons 12 and 13 present other mutations either in *KRAS*, *BRAF*, or *PIK3CA*, many of which may explain the lack of response to anti-EGFR therapy observed in a significant proportion of these patients.

## Competing interests

The authors declare that they have no competing interests.

## Authors’ contributions

JGG carried out most molecular genetic studies and drafted the manuscript. IV, PR, PP, CP, MP, and AP helped to set up, carry out, and interpret the molecular genetic studies. PL and RH provided histopathological data. MF, AR, PF, MM, NS, AA, JM, FA, CC and LLS provided patient clinical data. LLS and JGG performed the statistical analysis. MRT coordinated the study and helped to draft the manuscript. All authors read and approved the final manuscript.

## Pre-publication history

The pre-publication history for this paper can be accessed here:

http://www.biomedcentral.com/1471-2407/13/169/prepub

## Supplementary Material

Additional file 1 Clinicopathological features of the 201 patients. Click here for file

Additional file 2 Depiction of the PCR reaction mixture. Click here for file

Additional file 3 **Primer pairs used for *KRAS *mutation analysis and correspondent amplicon lengths.** Click here for file
