# Supplementary material for: High resolution melting analysis of KRAS, BRAF and PIK3CA in KRAS exon 2 wild-type metastatic colorectal cancer
Source: BMC Cancer. 2013 Apr 1;13:169. doi: 10.1186/1471-2407-13-169 (PMC3623853; doi:10.1186/1471-2407-13-169)
Supplement: Additional file 1 — Clinicopathological features of the 201 patients. [file 1471-2407-13-169-S1.pdf]

Supplementary Table 1 – Clinicopathological features of the 201 patients.

Case	Sex	Age at diagnosis	Location of the primary tumor	Anti-EGFR therapy
1	Male	28	Transverse colon	Yes
2	Female	33	Left colon	No
3	Female	35	Sigmoid colon	Yes
4	Female	35	Rectum	No
5	Male	36	Rectum	Yes
6	Female	37	Sigmoid colon	No
7	Male	39	Transverse colon	Yes
8	Male	41	Rectum	Yes
9	Female	41	Rectum	No
10	Male	43	Sigmoid colon	No
11	Male	44	Sigmoid colon	No
12	Female	45	Rectum	Yes
13	Female	46	Left colon	Yes
14	Female	47	Sigmoid colon	Yes
15	Male	47	Right colon	No
16	Female	48	Left colon	No
17	Female	49	Rectum	Yes
18	Female	49	Rectum	Yes
19	Male	49	Rectum	No
20	Male	50	Rectum	Yes
21	Female	50	Rectum	Yes
22	Female	50	Rectum	No
23	Male	50	Sigmoid colon	No
24	Female	50	Rectum	Yes
25	Male	50	Sigmoid colon	Yes
26	Female	50	Rectum	No
27	Male	50	Left colon	No
28	Male	50	Left colon	Yes

29	Male	50	Left colon	Yes
30	Female	51	Rectum	No
31	Male	51	Right colon	Yes
32	Male	52	Sigmoid colon	No
33	Male	52	Rectum	Yes
34	Male	52	Rectum	No
35	Male	53	Rectum	No
36	Female	53	Sigmoid colon	Yes
37	Male	53	Left colon	Yes
38	Male	54	Rectum	Yes
39	Male	54	Left colon	Yes
40	Female	54	Left colon	No
41	Male	55	Sigmoid colon	Yes
42	Female	55	Sigmoid colon	No
43	Female	55	Sigmoid colon	No
44	Female	55	Rectum	Yes
45	Female	55	Left colon	Yes
46	Male	55	Left colon	Yes
47	Female	56	Sigmoid colon	No
48	Male	56	Sigmoid colon	Yes
49	Male	56	Rectum	Yes
50	Female	56	Right colon	No
51	Female	57	Rectum	Yes
52	Male	57	Rectum	Yes
53	Male	57	Rectum	Yes
54	Male	57	Sigmoid colon	Yes
55	Male	57	Sigmoid colon	Yes
56	Male	57	Rectum	No
57	Male	57	Rectum	Yes
58	Male	57	Right colon	Yes
59	Female	58	Rectum	No

60	Male	58	Sigmoid colon	Yes
61	Male	58	Rectum	Yes
62	Male	58	Rectum	No
63	Male	58	Rectum	Yes
64	Male	58	Right colon	No
65	Female	58	Left colon	No
66	Male	59	Sigmoid colon	Yes
67	Male	59	Rectum	No
68	Male	59	Sigmoid colon	No
69	Male	59	Rectum	Yes
70	Female	59	Right colon	Yes
71	Male	59	Right colon	No
72	Male	59	Transverse colon	No
73	Male	60	Rectum	Yes
74	Male	60	Sigmoid colon	Yes
75	Male	60	Sigmoid colon	No
76	Male	60	Transverse colon	Yes
77	Male	60	Transverse colon	Yes
78	Male	61	Rectum	Yes
79	Male	61	Sigmoid colon	Yes
80	Female	61	Rectum	Yes
81	Male	61	Rectum	No
82	Female	61	Left colon	Yes
83	Male	62	Sigmoid colon	No
84	Female	62	Sigmoid colon	Yes
85	Male	62	Rectum	Yes
86	Male	62	Sigmoid colon	Yes
87	Male	62	Rectum	Yes
88	Male	62	Rectum	Yes
89	Male	63	Sigmoid colon	No
90	Female	63	Sigmoid colon	Yes

91	Female	63	Sigmoid colon	Yes
92	Female	63	Rectum	Yes
93	Male	63	Transverse colon	Yes
94	Male	63	Left colon	No
95	Male	64	Sigmoid colon	No
96	Male	64	Sigmoid colon	Yes
97	Male	64	Rectum	No
98	Female	64	Rectum	Yes
99	Male	64	Rectum	Yes
100	Male	64	Rectum	No
101	Male	64	Right colon	Yes
102	Male	65	Rectum	No
103	Male	65	Sigmoid colon	Yes
104	Female	65	Rectum	Yes
105	Male	65	Rectum	Yes
106	Female	65	Rectum	Yes
107	Male	65	Right colon	No
108	Male	65	Right colon	No
109	Female	65	Left colon	No
110	Male	66	Rectum	No
111	Male	66	Left colon	Yes
112	Male	66	Left colon	Yes
113	Male	67	Rectum	Yes
114	Male	67	Rectum	Yes
115	Male	67	Rectum	No
116	Female	69	Sigmoid colon	Yes
117	Male	69	Sigmoid colon	Yes
118	Male	69	Rectum	Yes
119	Male	69	Transverse colon	Yes
120	Male	70	Rectum	Yes
121	Female	70	Rectum	Yes

122	Female	72	Rectum	No
123	Female	73	Sigmoid colon	No
124	Female	74	Sigmoid colon	No
125	Male	76	Sigmoid colon	No
126	Male	56	Rectum	No
127	Female	83	Colon	No
128	Female	63	Rectum	No
129	Male	55	Left colon	Yes
130	Male	71	Sigmoid colon	No
131	Male	77	Left colon	No
132	Male	59	Rectum	No
133	Male	64	Sigmoid colon	No
134	Male	63	Rectum	Yes
135	Male	80	Colon	No
136	Male	39	Rectum	No
137	Male	40	Sigmoid colon	No
138	Male	58	Rectum	No
139	Male	58	Right colon	No
140	Male	83	Sigmoid colon	No
141	Female	59	Right colon	No
142	Male	63	Rectum	No
143	Male	84	Colon	No
144	Male	69	Sigmoid colon	No
145	Male	59	Colon	Yes
146	Male	68	Colon	No
147	Male	57	Sigmoid colon	No
148	Female	55	Sigmoid colon	No
149	Male	67	Rectum	Yes
150	Female	78	Sigmoid colon	Yes
151	Male	82	Sigmoid colon	No
152	Male	70	Sigmoid colon	No

153	Male	51	Sigmoid colon	No
154	Male	77	Sigmoid colon	Yes
155	Male	80	Right colon	Yes
156	Male	71	Rectum	No
157	Female	64	Rectum	No
158	Male	39	Sigmoid colon	No
159	Male	64	Rectum	Yes
160	Male	65	Sigmoid colon	Yes
161	Male	79	Right colon	No
162	Male	60	Sigmoid colon	No
163	Male	58	Rectosigmoid junction	No
164	Male	71	Rectum	No
165	Female	79	Cecum	No
166	Male	58	Cecum	Yes
167	Female	64	Right colon	No
168	Male	84	Rectum	No
169	Female	71	Sigmoid colon	No
170	Female	71	Transverse colon	No
171	Female	45	Sigmoid colon	Yes
172	Female	79	Rectum	No
173	Male	75	Right colon	Yes
174	Female	73	Rectum	No
175	Female	48	Sigmoid colon	Yes
176	Male	77	Sigmoid colon	No
177	Male	71	Sigmoid colon	No
178	Male	89	Right colon	No
179	Male	68	Sigmoid colon	No
180	Male	78	Rectum	No
181	Male	54	Sigmoid colon	No
182	Male	72	Sigmoid colon	No
183	Male	68	Left colon	No

184	Male	49	Rectum	Yes
185	Male	70	Sigmoid colon	No
186	Male	45	Rectum	No
187	Male	48	Transverse colon	Yes
188	Female	68	Left colon	No
189	Male	72	Left colon	No
190	Female	65	Right colon	No
191	Male	62	Right colon	Yes
192	Male	74	Rectum	No
193	Male	53	Rectum	No
194	Female	66	Sigmoid colon	No
195	Male	68	Rectum	Yes
196	Male	60	Rectum	No
197	Male	68	Transverse colon	No
198	Male	65	Sigmoid colon	No
199	Female	64	Transverse colon	Yes
200	Female	79	Sigmoid colon	No
201	Male	75	Rectum	Yes
