# Supplementary material for: High resolution melting analysis of KRAS, BRAF and PIK3CA in KRAS exon 2 wild-type metastatic colorectal cancer
Source: BMC Cancer. 2013 Apr 1;13:169. doi: 10.1186/1471-2407-13-169 (PMC3623853; doi:10.1186/1471-2407-13-169)
Supplement: Additional file 2 — Depiction of the PCR reaction mixture. [file 1471-2407-13-169-S2.pdf]

Supplementary Table 2 – Depiction of the PCR reaction mixture.

	PIK3CA **exon 9**	***PIK3CA*** **exon 20**	***BRAF*** **exon 11**	***BRAF*** **exon 15**	***KRAS*** **exon 3**	***KRAS*** **exon 4**
PCR reaction components						
2,5x LightScanner® Master Mix [Idaho]	1x	1x	1x	1x	1x	1x
Reagent grade water [Idaho] (µL)	4,4	4,4	4,4	4,4	4,4	4,1
Forward primer (nM)	300	300	300	300	300	200
Reverse primer (nM)	300	300	300	300	300	200
DNA (ng)	20 -100	20 -100	20 -100	20 -100	20 -100	20 -100
MgCl2 (mM)	-	-	-	-	-	0,5
Total reaction volume (µL)	10	10	10	10	10	10
