# Supplementary material for: High resolution melting analysis of KRAS, BRAF and PIK3CA in KRAS exon 2 wild-type metastatic colorectal cancer
Source: BMC Cancer. 2013 Apr 1;13:169. doi: 10.1186/1471-2407-13-169 (PMC3623853; doi:10.1186/1471-2407-13-169)
Supplement: Additional file 3 — Primer pairs used for KRAS mutation analysis and correspondent amplicon lengths. [file 1471-2407-13-169-S3.pdf]

Supplementary Table 3 – Primer pairs used for *KRAS* mutation analysis and correspondent amplicon lengths.

***KRAS* exon 3**	
Forward primer sequence	5' CCA GAC TGT GTT TCT CCC TTC TCA GG 3'
Reverse primer sequence	5' AGA AAG CCC TCC CCA GTC CTC A 3'
Amplicon length	150 bp
***KRAS* exon 4**	
Forward primer sequence	5' ACAGGCTCAGGACTTAGCAAGAAGT 3'
Reverse primer sequence	5' AGCATAATTGAGAGAAAAACTGA 3'
Amplicon length	158 bp
